# N-acetylcysteine supplementation for the prevention of atrial fibrillation after cardiac surgery: a meta-analysis of eight randomized controlled trials

**DOI:** 10.1186/1471-2261-12-10

**Published:** 2012-02-24

**Authors:** Wan-Jie Gu, Zhen-Jie Wu, Peng-Fei Wang, Lynn Htet Htet Aung, Rui-Xing Yin

**Affiliations:** 1 Department of Cardiology, Institute of Cardiovascular Diseases, the First Affiliated Hospital, Guangxi Medical University, 22 Shuangyong Road, Nanning 530021, Guangxi, People's Republic of China; 2 Department of Colorectal and Anal Surgery, the First Affiliated Hospital, Guangxi Medical University, Nanning, Guangxi, People's Republic of China; 3 Department of Orthopaedics, China-Japan Union Hospital, Jilin University, Changchun, Jilin, People's Republic of China

## Abstract

**Background:**

Atrial fibrillation is the most common type of arrhythmia after cardiac surgery. An increasing body of evidence demonstrates that oxidative stress plays a pivotal role in the pathophysiology of atrial fibrillation. N-acetylcysteine (NAC) is a free radical scavenger, and may attenuate this pathophysiologic response and reduce the incidence of postoperative AF (POAF). However, it is unclear whether NAC could effectively prevent POAF. Therefore, this meta-analysis aims to assess the efficacy of NAC supplementation on the prevention of POAF.

**Methods:**

Medline and Embase were systematically reviewed for studies published up to November 2011, in which NAC was compared with controls for adult patients undergoing cardiac surgery. Outcome measures comprised the incidence of POAF and hospital length of stay (LOS). The meta-analysis was performed with the fixed-effect model or random-effect model according to the heterogeneity.

**Results:**

Eight randomized trials incorporating 578 patients provided the best evidence and were included in this meta-analysis. NAC supplementation significantly reduced the incidence of POAF (OR 0.62, 95% CI 0.41 to 0.93; *P *= 0.021) compared with controls, but had no effect on LOS (WMD -0.07, 95% CI -0.42 to 0.28; *P *= 0.703).

**Conclusions:**

The prophylactic NAC supplementation may effectively reduce the incidence of POAF. However, the overall quality of current studies is poor and further research should focus on adequately powered randomized controlled trials with POAF incidence as a primary outcome measure.

## Background

Postoperative atrial fibrillation (POAF) is the most frequent arrhythmia encountered following cardiac surgery, affecting approximately 25-40% of patients [1-3]. Although the majority of POAF are benign and self-limiting, it has been associated with an increase in both hospital length of stay (LOS) and total hospital costs [1,2,4,5]. The efficacy of pharmacologic interventions on preventing POAF has been extensively researched [6,7]. Recent guidelines for the prevention and management of POAF were published in 2011 jointly by the American College of Cardiology, the AHA, and the European Society of Cardiology [8]. Nevertheless, none of them are effective for all patients and all of them have significant limitations, the best prophylaxis to prevent POAF remains to be established [9]. Despite the extensive studies, the pathphysiology of POAF are for the moment far from being fully elucidated. In the past few years, a growing body of evidence suggests that oxidative stress has been found to play a pivotal role in the pathophysiology of POAF [10]. Oxidative stress may be one of a possible pathogenesis of POAF [11].

Antioxidant N-acetylcysteine (NAC) seemed a promising and novel measure for the prevention of POAF. In a prospective, randomized, placebo-controlled pilot study in patients undergoing coronary artery bypass and/or valve surgery, NAC significantly reduced the incidence of POAF [12]. However, the results have contrasted in seven other randomized controlled trials [13-19] in which the effectiveness of NAC was assessed in patients undergoing cardiac surgery. We therefore conducted a meta-analysis based on relevant and available randomized controlled trials to assess the efficacy of NAC supplementation on the prevention of POAF for adult patients undergoing cardiac surgery.

## Methods

### Literature search and selection criteria

A comprehensive search was undertaken to identify all published randomized controlled trials of NAC versus controls during cardiac surgery. Medline and Embase were searched from the date of their inception to November 1, 2011. Search terms included: "N-acetylcysteine", "NAC", "acetylcysteine", "mucomyst" and "cardiac surgery", "cardiothoracic surgery","heart surgery", "cardiopulmonary bypass", "CPB", "coronary artery bypass graft", "CABG", "CAB", "valve surgery", and "valvular surgery". Results were limited to human subjects and randomized controlled trials. We screened the reference lists of included studies and related publications. The results were then hand searched for eligible trials. We did not include abstracts or meeting's proceedings. This search strategy was performed iteratively until no new potential citations could be found on review of the reference lists of retrieved articles.

We included studies in all languages irrespective of blinding when the following inclusion criteria were met: adult patients undergoing cardiac surgery; randomized allocation to NAC group or control group (placebo or routine treatment); and reporting data on the incidence of POAF. Exclusion criteria included age < 18 years old, and known allergy or hypersensitivity to NAC.

### Data extraction and quality assessment

Two investigators (Wan-Jie Gu and Zhen-Jie Wu) independently extracted the following information from each study: first author's name, surgery type (coronary artery bypass graft [CABG], valve, or combination surgery), study design (RCT, prospective or not), type of controls (placebo or not), type of blinding (double-blinding or not), NAC regimen, sample size, mean age, percentage male, the incidence of POAF, and length of hospital stay in each group. Disagreements were resolved through discussion and consensus.

The methodological quality of the studies included in the meta-analysis was independently scored by Wan-Jie Gu and Zhen-Jie Wu using validated Jadad 5 point scale. The scale consists of three items describing randomization (0-2 points), masking (0-2 points), and dropouts and withdrawals (0-1 points) in the report of a randomized controlled trial [20]. A score of 1 is given for each of the points described. A further point is obtained where the method of randomization and/or blinding is given and is appropriate; whereas it is inappropriate a point is deducted. Higher scores indicate better reporting.

### Statistical analysis

We assessed the overall efficacy of NAC supplementation on the prevention of POAF based on the data from the eight randomized trials. The incidences of POAF were treated as dichotomous variables and were expressed as odds ratio (OR) with 95% CI for each study. LOS was treated as a continuous variable. For comparison of LOS, the weighted mean difference (WMD) with 95% CI was calculated as the difference between the mean values of LOS in treatment and control groups. Pooled estimates of efficacy were calculated using the Man-tel-Haenszel fixed-effects model [21]. If there was heterogeneity, the following methods were used to deal with it: (a) subgroup analysis; (b) sensitivity analysis performed by excluding the trials which potentially biased the results. If the heterogeneity still potentially existed, the DerSimonian and Lair random-effects model was used. A test for heterogeneity, defined as variation among the results of individual trials for a given treatment beyond that expected from chance, was used to assess whether the magnitude of a given treatment effect varied between the trials. We assessed heterogeneity with I^2^, which describes the percentage of total variation across studies due to heterogeneity rather than chance. I^2 ^can be calculated as: I^2 ^= 100% × (Q-df)/Q(Q = Cochrane's heterogeneity statistics, df = degrees of freedom). Negative values of I^2 ^equaled zero, so that I^2 ^ranged between 0% (ie, no observed heterogeneity) and 100%. High values would show increasing heterogeneity [22]. The presence of publication bias was evaluated by using the Begg and Egger tests [23,24]. A two-tailed *P*-value of less than 0.05 was judged as statistically significant. All statistical analyses were performed using Stata version 11 (Stata Corporation, College Station, TX, USA).

## Results

### Identification of eligible studies

**Figure 1:**
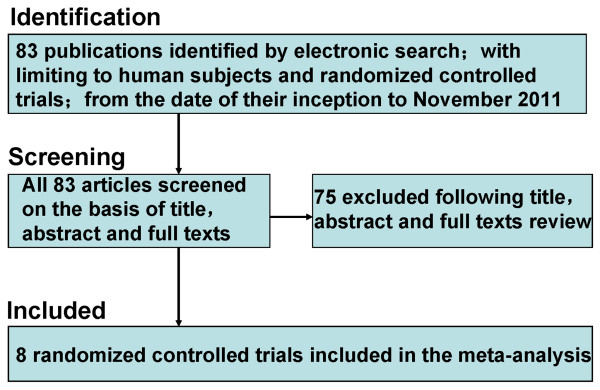
**Search strategy**.

Eighty-three reports were identified by the initial literature retrieval, with limiting to human subjects and randomized controlled trials. Through various means, we accessed all the full texts. After screening the title, abstract and full texts, however, 75 studies were excluded because they did not provide available data. Consequently, the remaining 8 trials (n = 578 patients) provided best evidence and were included in the current meta-analysis [12-19]. The flow chart of search strategy is shown in Figure 1.

### Characteristics of eligible studies

The baseline characteristics of the included studies are shown in Table 1 and design characteristics are presented in Table 2. Of the 8 included trials, four were done in Turkey [12-14,18], two in Canada [15,17], one in Germany [16], and one in Korea [19]. The number of participants ranged from 20 [13,14] to 175 [17]. All trials included both men and women. Five studies [13-15,18,19] in this meta-analysis enrolled patients undergoing CABG only. The remaining three studies [12,16,17] included patients undergoing valvular or combination CABG with valvular surgery, or both. NAC was administered orally and/or intravenously by different regimens and formulations. Seven studies [12-14,16-19] used an intravenous route to administer NAC during the perioperative period, and one study [15] used an oral dose before surgery followed by intravenous administration postoperatively. The overwhelming majority of studies used weight-based dosing and started NAC just a few hours prior to surgery. The overall incidence of POAF varied between 5% [14] and 61.7% [17]. A total of 289 patients were allocated randomly to NAC group and 289 to control group. The quality of the included studies was assessed by the Jadad score. The median Jadad score of the studies included was 4 (range from 3 to 5).

**Table 1:** Baseline characteristics of the included studies

Reference	NAC regimen	N		n	Age (years)		Male (%)	
					
			NAC	Control	NAC	Control	NAC	Control
Ozaydin et al. [12]	50 mg/kg IV 1 h before surgery and 50 mg/kg/day for 48 h post-CTS	115	58	57	57 ± 11	59 ± 9	81	77.2
Eren et al. [13]	100 mg/kg IV for 1 h before CPB and 40 mg/kg/day at 24 h after CPB	20	10	10	61.1 ± 4.8	60.5 ± 5.7	80	70
Orhan et al. [14]	50 mg/kg IV at start of anesthesia induction for 30 min	20	10	10	59.6 ± 5.48	61.8 ± 4.32	70	60
El-Hamamsy et al. [15]	600 mg orally the day before and the morning of the operation,150 mg/kg IV before skin incision, then 12.5 mg/kg/h IV for 24 h	100	50	50	59.8 ± 7.8	61.3 ± 7.4	86	92
Haase et al. [16]#	150 mg/kg IV bolus after anesthesia induction, then 50 mg/kg IV over 4 h, then 100 mg/kg IV over 20 h	60	30	30	68.9 ± 9.7	68.3 ± 9.3	77	70
Wijeysundera et al. [17]	100 mg/kg IV after induction of anesthesia over 30 min, 20 mg/kg/h IV after CPB for 4 h	175	88	87	74 ± 8	73 ± 9	60	59
Peker et al. [18]	50 mg/kg IV for 1 h before surgery, 50 mg/kg/day IV for 48 h after the operation	40	19	21	60.00 ± 11.36	57.67 ± 8.57	89.5	85.7
Kim et al. [19]	100 mg/kg IV bolus over 15 min after anesthetic induction, then IV infusion at 40 mg/kg/day for 24 h	48	24	24	60.8 ± 8.4	65.3 ± 7.6	87.5	91.7

Abbreviations: NAC, N-acetylcysteine; CPB, cardiopulmonary bypass; IV, intravenous; CTS, cardiothoracic surgery; #, history of AF; N, Number of participants

**Table 2:** Design characteristics

						POAF		LOS(days)	
						
Reference	Surgery type	Study design	Control group	Double- blinding	Jadad Score	NAC	Control	NAC	Control
Ozaydin et al. [12]	CABG and/or valve	RCT (prospective)	Placebo	Yes	4	3/58	12/57	7.7 ± 3	7.9 ± 4.2
Eren et al. [13]	CABG	RCT (prospective)	Placebo	Yes	3	2/10	1/10	NA	NA
Orhan et al. [14]	CABG	RCT	Routine protocol	No	3	0/10	1/10	7.2 ± 0.42	7.3 ± 0.48
El-Hamamsy et al. [15]	CABG	RCT (prospective)	Placebo	Yes	3	4/50	6/50	5.4 ± 2.3	5.3 ± 2.5
Haase et al. [16]#	CABG and/or valve	RCT	Placebo	Yes	5	19/30	16/30	8(7-11)	8(7-11)
Wijeysundera et al. [17]	CABG and/or valve	RCT	Placebo	Yes	5	50/88	58/87	NA	NA
Peker et al. [18]	CABG	RCT (prospective)	Placebo	Yes	4	0/19	2/21	NA	NA
Kim et al. [19]	CABG	RCT	Placebo	Yes	4	4/24	8/24	11.3 ± 6.3	10.5 ± 4.5

Abbreviations: NAC, N-acetylcysteine; POAF, postoperative atrial fibrillation; LOS, length of hospital stay; CABG, coronary artery bypass grafting; #, history of AF; RCT, randomized controlled trial; NA, data not available

### POAF

POAF data were reported in all eight studies [12-19] (578 patients; Table 2), and POAF was the primary end point in one study [12]. Details of POAF outcome definition and assessment are presented in Table 3.

**Table 3:** Monitoring and definition of postoperative atrial fibrillation

Reference	Monitoring of postoperative atrial fibrillation	Definition of postoperative atrial fibrillation
Ozaydin et al. [12]	ECGs performed continuously during the first 2 postoperative days in the intensive care unit	An irregular narrow complex rhythm (in the absence of bundle branch block) with absence of discrete P-waves
Eren et al. [13]	ECGs performed on the first postoperative day	Not reported
Orhan et al. [14]	Not reported	Not reported
El-Hamamsy et al. [15]	Not reported	Not reported
Haase et al. [16]	Not reported	Not reported
Wijeysundera et al. [17]	Continuous telemetry or 12-lead ECGs	Any new atrial fibrillation
Peker et al. [18]	ECGs performed continuously during the first 2 postoperative days in the intensive care unit	Not reported
Kim et al. [19]	Not reported	Not reported

ECG, electrocardiogram

Analysis of pooled prevalence of preoperative patient group characteristics revealed that no difference was observed for history of coexistence of basic diseases (e.g. diabetes mellitus, hypertension, congestive heart failure), routine prophylactic therapies (e.g. β-blocker, angiotensin-converting enzyme inhibitor or angiotensin receptor blocker, and calcium channel blocker; Table 4).

**Table 4:** Perioperative variables of the patients

Variable	NAC [% (n)]	Control [% (n)]	*X*^2 ^value	*P *value	Total prevalence [% (n)]
Diabetes mellitus [12,14,16,17,19]	33.8 (71/210)	30.8 (64/208)	0.442	0.506	32.3 (135/418)
Hypertension [12,14,16,17,19]	70.5 (148/210)	69.7 (145/208)	0.029	0.864	70.1 (293/418)
Congestive heart failure [17]	19.3 (17/88)	18.4 (16/87)	0.025	0.875	18.9 (33/175)
β-blocker [12,15,17,19]	75.9 (167/220)	73.4 (160/218)	0.366	0.545	74.7 (327/438)
ACEI or ARB [12,15,17,19]	64.1 (141/220)	60.1 (131/218)	0.744	0.388	62.1 (272/438)
Calcium channel blockers [15,17,19]	40.1 (65/162)	34.8 (56/161)	0.983	0.321	37.5 (121/323)

ACEI, angiotensin-converting enzyme inhibitor; ARB, angiotensin receptor blocker

**Figure 2:**
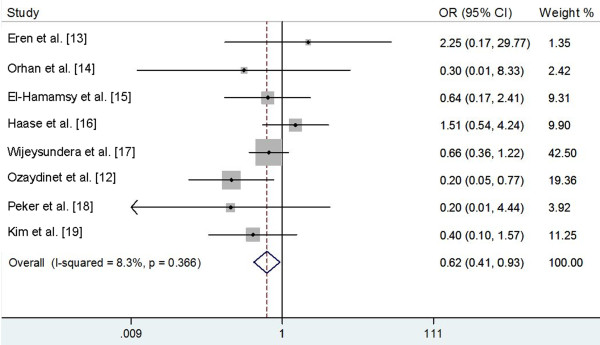
**Incidence of postoperative atrial fibrillation (fixed effect model)**. CI, confidence interval; OR, odds ratio.

Pooling all eight randomized trials, 28.4% (82 of 289) of patients given NAC and 36.0% (104 of 289) of controls developed POAF. Meta-analysis of eight trials using a fixed-effects model showed that NAC supplementation significantly reduced the incidence of POAF (OR 0.62, 95% CI 0.41 to 0.93; *P *= 0.021; Figure 2) compared with controls, with little statistical heterogeneity between the trials (I^2 ^= 8.3%, heterogeneity *P *= 0.366).

A trial conducted by Haase et al. [16] reported some patients with pre-existing atrial fibrillation. The pooled results didn't significantly change when this trial was excluded (OR 0.52, 95% CI 0.33 to 0.82; *P *= 0.005), and no evidence of heterogeneity was observed among the remaining seven studies (I^2 ^= 0.0%, heterogeneity *P *= 0.622).

### Hospital LOS

**Figure 3:**
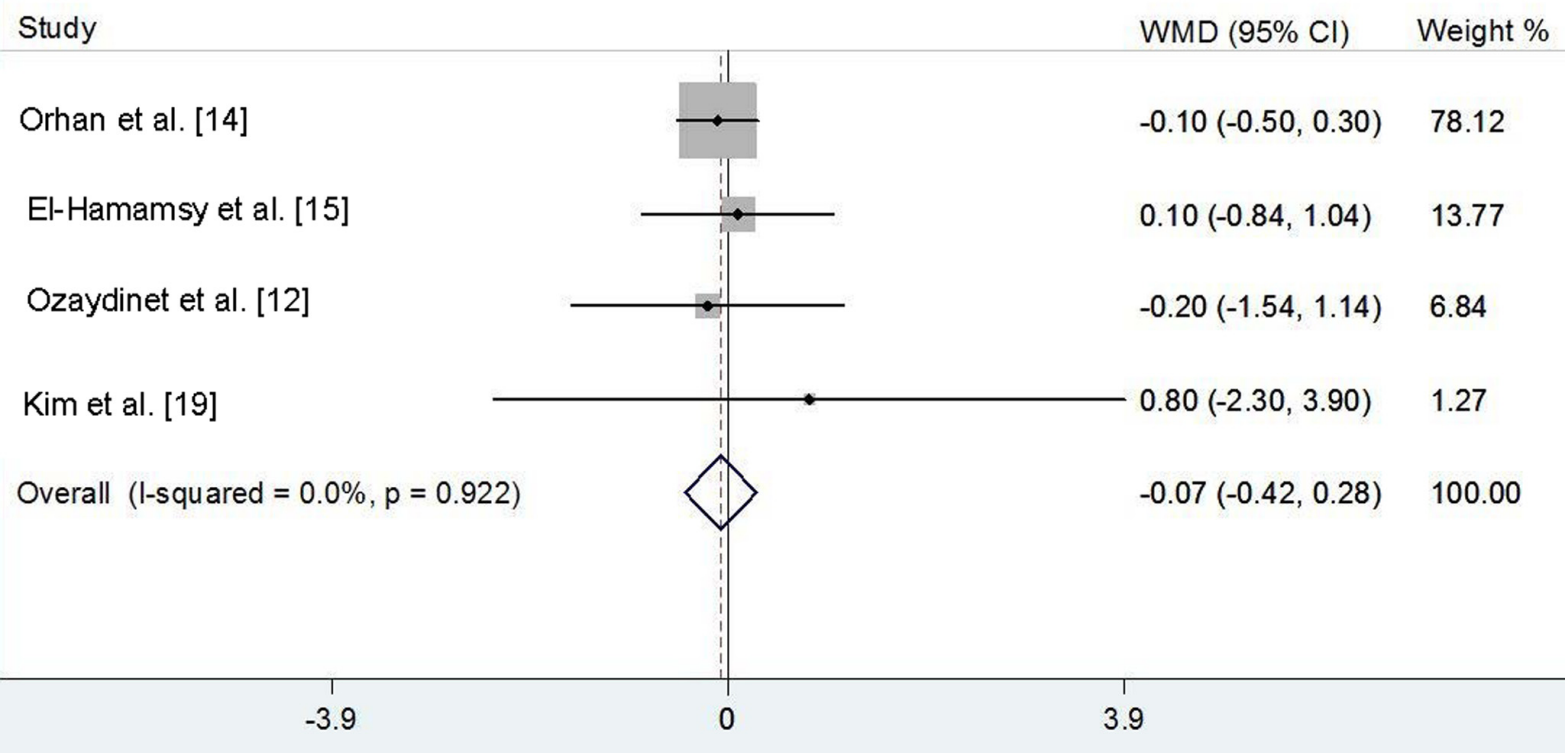
**Effects of N-acetylcysteine on hospital length of stay (days)**. WMD, weighted mean difference.

Five trials [12,14-16,19] reported the effect of NAC on LOS, but only 4 trials [12,14,15,19] provided available data (expressed as mean ± standard deviation) with a total of 283 patients. The pooled analysis using a fixed-effects model showed that NAC supplementation did not significantly reduce LOS (WMD -0.07, 95% CI -0.42 to 0.28; *P *= 0.703; Figure 3), with no statistical heterogeneity between the trials (I^2 ^= 0.0%, heterogeneity *P *= 0.922).

### Publication bias

**Figure 4:**
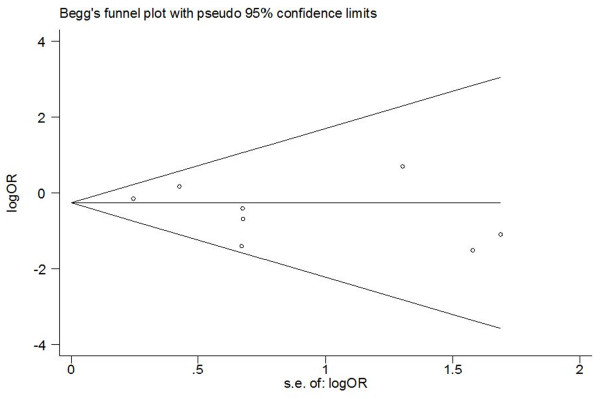
**Tests for publication bias for OR of the incidence of POAF**.

Assessment of publication bias using Egger's and Begg's tests showed that there was no potential publication bias among the included trials (Egger's test, *P *= 0.275; Begg's test, *P *= 0.536, Figure 4).

## Discussion

To the best of our knowledge, this is the first meta-analysis to assess the efficacy of NAC supplementation on the prevention of atrial fibrillation after cardiac surgery. Meta-analysis of all eight included trials using a fixed-effects model illustrates that NAC supplementation can effectively reduce the incidence of POAF in adult patients undergoing cardiac surgery. In a recent meta-analysis, Harling et al. [5] evaluated antioxidant vitamins as a prophylactic method against POAF. This intervention reduced POAF significantly with OR of 0.43 (95% CI 0.21 to 0.89). Our results suggest that antioxidant NAC supplementation is as effective as antioxidant vitamins in reducing POAF, with OR in the similar range, being vitamins slightly more effective achieving this purpose than NAC. In addition, one problem with the use of antioxidant NAC to prevent POAF is that the majority of patients does not develop POAF after cardiac surgery but would still be exposed to possible side effects. In this meta-analysis, three trials [12,17,18] reported NAC-related adverse effects and the incidence of postoperative complications was similar in both NAC and control groups.

The mechanisms that antioxidant NAC supplementation reduces the incidence of POAF are not entirely known. However, there is now an increasing body of evidences that oxidative stress [11], inflammation [25,26], and rennin-angiotensin system [27,28] are involved in the pathogenesis of POAF.

The potential role of oxidative stress in the genesis and perpetuation of POAF is an interesting subject. NAC is a glutathione precursor; by entering cells and being hydrolyzed to cysteine, it stimulates glutathione synthesis, scavenges free radicals, and terminates the propagation of free radical reactions [29]. In addition, it also has potent anti-inflammatory effects by reducing the production of pro-inflammatory cytokines [30,31] and blocks rennin-angiotensin system and/or atrial remodelling via its anti-inflammatory and antioxidant actions [32].

Aranki et al. [33] demonstrated that atrial fibrillation is a major predictor of longer hospitalization and found that it was independently associated with LOS extended by 4.9 days. NAC supplementation did not significantly reduce the LOS in this meta-analysis. The total incidence of POAF is 32.2% (156 of 578), fewer than half of patients develop POAF and still fewer develop prolonged atrial fibrillation, so the effect of NAC on LOS in patients prone to atrial fibrillation would have to be very large to be able to detect an effect of LOS in the total population. In addition, a relatively small number of samples (only four studies) provided available data on LOS, additional studies or data are warranted.

This meta-analysis has several potential limitations that should be taken into account. First, we acknowledged that the meta-analysis included the relatively small number of patients in the individual studies (n < 50 in four studies [13,14,18,19]) and is limited by the lack of complete availability of relevant data, particularly for LOS. Second, only one study [12] included in the present meta-analysis has used POAF as a primary outcome measure, but for the remaining seven studies [13-19], POAF is only one of the clinically significant end points consistently reported rather than a primary outcome measure. Next, these studies lack homogeneity in both the method of postoperative monitoring and in their definition of POAF. This leads to potential underestimation and/or overestimation of the true incidence of POAF. Finally, because of sparse and inconsistent reporting across trials, we were unable to assess the impact of variations in the use of routine prophylactic therapies (e.g. statins, diuretics, NSAID or COX-2 inhibitor, and aspirin etc.), coexistence of basic diseases (e.g. myocardial infarction etc.), and other factors on our meta-analytic conclusions.

Future research should therefore focus on an adequately powered multicentre large-scale double-blind placebo-controlled randomized trial. Furthermore, novel studies should aim to treat the incidence of POAF as a primary outcome measure, and standardize POAF reporting criteria and antioxidant protocol. It is also need to exclude the synergistic effects of other coexisted drugs and antioxidant NAC on POAF.

## Conclusion

This meta-analysis indicates that prophylactic NAC supplementation effectively reduces the incidence of POAF for adult patients undergoing cardiac surgery. Further research should focus on adequately powered RCTs with POAF incidence as a primary outcome measure.

## Competing interests

The authors declare that they have no competing interests.

## Authors' contributions

WJG conceived the study, participated in the design, collected the data, and drafted the manuscript. ZJW collected the data, and performed statistical analyses. PFW and LHHA helped to collect the data. RXY conceived the study, participated in the design, and helped to draft the manuscript. All authors read and approved the final manuscript.

## Pre-publication history

The pre-publication history for this paper can be accessed here:

http://www.biomedcentral.com/1471-2261/12/10/prepub
